# A scoping review to identify and organize literature trends of bias research within medical student and resident education

**DOI:** 10.1186/s12909-023-04829-6

**Published:** 2023-12-05

**Authors:** Brianne E. Lewis, Akshata R. Naik

**Affiliations:** 1 Department of Foundational Sciences, Central Michigan University College of Medicine, Mt. Pleasant, MI 48859 USA; 2 Department of Foundational Medical Studies, Oakland University William Beaumont School of Medicine, 586 Pioneer Dr, Rochester, MI 48309 USA

**Keywords:** Bias, Medical student, Resident, Preclinical curriculum, Evidence of bis, Bias intervention

## Abstract

**Background:**

Physician bias refers to the unconscious negative perceptions that physicians have of patients or their conditions. Medical schools and residency programs often incorporate training to reduce biases among their trainees. In order to assess trends and organize available literature, we conducted a scoping review with a goal to categorize different biases that are studied within medical student (MS), resident (Res) and mixed populations (MS and Res). We also characterized these studies based on their research goal as either documenting evidence of bias (EOB), bias intervention (BI) or both. These findings will provide data which can be used to identify gaps and inform future work across these criteria.

**Methods:**

Online databases (PubMed, PsycINFO, WebofScience) were searched for articles published between 1980 and 2021. All references were imported into Covidence for independent screening against inclusion criteria. Conflicts were resolved by deliberation. Studies were sorted by goal: ‘evidence of bias’ and/or ‘bias intervention’, and by population (MS or Res or mixed) andinto descriptive categories of bias.

**Results:**

Of the initial 806 unique papers identified, a total of 139 articles fit the inclusion criteria for data extraction. The included studies were sorted into 11 categories of bias and showed that bias against race/ethnicity, specific diseases/conditions, and weight were the most researched topics. Of the studies included, there was a higher ratio of EOB:BI studies at the MS level. While at the Res level, a lower ratio of EOB:BI was found.

**Conclusions:**

This study will be of interest to institutions, program directors and medical educators who wish to specifically address a category of bias and identify where there is a dearth of research. This study also underscores the need to introduce bias interventions at the MS level.

## Background

Physician bias ultimately impacts patient care by eroding the physician–patient relationship [1–4]. To overcome this issue, certain states require physicians to report a varying number of hours of implicit bias training as part of their recurring licensing requirement [5, 6]. Research efforts on the influence of implicit bias on clinical decision-making gained traction after the “Unequal Treatment: Confronting Racial and Ethnic Disparities in Health Care” report published in 2003 [7]. This report sparked a conversation about the impact of bias against women, people of color, and other marginalized groups within healthcare. Bias from a healthcare provider has been shown to affect provider-patient communication and may also influence treatment decisions [8, 9]. Nevertheless, opportunities within medical education curriculum are created to evaluate biases at an earlier stage of physician-training and provide instruction to intervene them [10–12]. We aimed to identify trends and organize literature on bias training provided during medical school and residency programs since the meaning of ‘bias’ is broad and encompasses several types of attitudes and predispositions [13].

Several reviews, narrative or systematic in nature, have been published in the field of bias research in medicine and healthcare [14–16]. Many of these reviews have a broad focus on implicit bias and they often fail to define the patient’s specific attributes- such as age, weight, disease, or condition against which physicians hold their biases. However, two recently published reviews categorized implicit biases into various descriptive characteristics albeit with research goals different than this study [17, 18]. The study by Fitzgerald et al. reviewed literature focused on bias among physicians and nurses to highlight its role in healthcare disparities [17]. While the study by Gonzalez et al. focused on bias curricular interventions across professions related to social determinants of health such as education, law, medicine and social work [18]. Our research goal was to identify the various bias characteristics that are studied within medical student and/or resident populations and categorize them. Further, we were interested in whether biases were merely identified or if they were intervened. To address these deficits in the field and provide clarity, we utilized a scoping review approach to categorize the literature based on a) the bias addressed and b) the study goal within medical students (MS), residents (Res) and a mixed population (MS and Res).

To date no literature review has organized bias research by specific categories held solely by medical trainees (medical students and/or residents) and quantified intervention studies. We did not perform a quality assessment or outcome evaluation of the bias intervention strategies, as it was not the goal of this work and is standard with a scoping review methodology [19, 20]. By generating a comprehensive list of bias categories researched among medical trainee population, we highlight areas of opportunity for future implicit bias research specifically within the undergraduate and graduate medical education curriculum. We anticipate that the results from this scoping review will be useful for educators, administrators, and stakeholders seeking to implement active programs or workshops that intervene specific biases in pre-clinical medical education and prepare physicians-in-training for patient encounters. Additionally, behavioral scientists who seek to support clinicians, and develop debiasing theories [21] and models may also find our results informative.

## Methods

We conducted an exhaustive and focused scoping review and followed the methodological framework for scoping reviews as previously described in the literature [20, 22]. This study aligned with the four goals of a scoping review [20]. We followed the first five out of the six steps outlined by Arksey and O’Malley’s to ensure our review’s validity 1) identifying the research question 2) identifying relevant studies 3) selecting the studies 4) charting the data and 5) collating, summarizing and reporting the results [22]. We did not follow the optional sixth step of undertaking consultation with key stakeholders as it was not needed to address our research question it [23]. Furthermore, we used Covidence systematic review software (Veritas Health Innovation, Melbourne, Australia) that aided in managing steps 2–5 presented above.

### Research question, search strategy and inclusion criteria

The purpose of this study was to identify trends in bias research at the medical school and residency level. Prior to conducting our literature search we developed our research question and detailed the inclusion criteria, and generated the search syntax with the assistance from a medical librarian. Search syntax was adjusted to the requirements of the database. We searched PubMed, Web of Science, and PsycINFO using MeSH terms shown below.

- Bias* [ti] OR prejudice*[ti] OR racism[ti] OR homophobia[ti] OR mistreatment[ti] OR sexism[ti] OR ageism[ti]) AND (prejudice [mh] OR "Bias"[Mesh:NoExp]) AND (Education, Medical [mh] OR Schools, Medical [mh] OR students, medical [mh] OR Internship and Residency [mh] OR “undergraduate medical education” OR “graduate medical education” OR “medical resident” OR “medical residents” OR “medical residency” OR “medical residencies” OR “medical schools” OR “medical school” OR “medical students” OR “medical student”) AND (curriculum [mh] OR program evaluation [mh] OR program development [mh] OR language* OR teaching OR material* OR instruction* OR train* OR program* OR curricul* OR workshop*

Our inclusion criteria incorporated studies which were either original research articles, or review articles that synthesized new data. We excluded publications that were not peer-reviewed or supported with data such as narrative reviews, opinion pieces, editorials, perspectives and commentaries. We included studies outside of the U.S. since the purpose of this work was to generate a comprehensive list of biases. Physicians, regardless of their country of origin, can hold biases against specific patient attributes [17]. Furthermore, physicians may practice in a different country than where they trained [24]. Manuscripts were included if they were published in the English language for which full-texts were available. Since the goal of this scoping review was to assess trends, we accepted studies published from 1980–2021.

Our inclusion criteria also considered the *goal* and the *population* of the study. We defined the study *goal* as either that documented evidence of bias or a program directed bias intervention. Evidence of bias (EOB) had to originate from the medical trainee regarding a patient attribute. Bias intervention (BI) studies involved strategies to counter biases such as activities, workshops, seminars or curricular innovations. The *population* studied had to include medical students (MS) or residents (Res) or mixed. We defined the study population as ‘mixed’ when it consisted of both MS and Res. Studies conducted on other healthcare professionals were included if MS or Res were also studied. Our search criteria excluded studies that documented bias against medical professionals (students, residents and clinicians) either by patients, medical schools, healthcare administrators or others, and was focused on studies where the biases were solely held by medical trainees (MS and Res).

### Data extraction and analysis

Following the initial database search, references were downloaded and bulk uploaded into Covidence and duplicates were removed. After the initial screening of title and abstracts, full-texts were reviewed. Authors independently completed title and abstract screening, and full text reviews. Any conflicts at the stage of abstract screening were moved to full-text screening. Conflicts during full-text screening were resolved by deliberation and referring to the inclusion and exclusion criteria detailed in the research protocol. The level of agreement between the two authors for full text reviews as measured by inter-rater reliability was 0.72 (Cohen’s Kappa).

A data extraction template was created in Covidence to extract data from included full texts. Data extraction template included the following variables; country in which the study was conducted, year of publication, goal of the study (EOB, BI or both), population of the study (MS, Res or mixed) and the type of bias studied. Final data was exported to Microsoft Excel for quantification. For charting our data and categorizing the included studies, we followed the Preferred Reporting Items for Systematic Reviews and Meta-Analyses extension for Scoping Reviews(PRISMA-ScR) guidelines [25]. Results from this scoping review study are meant to provide a visual synthesis of existing bias research and identify gaps in knowledge.

## Results

### Study selection

**Fig. 1:**
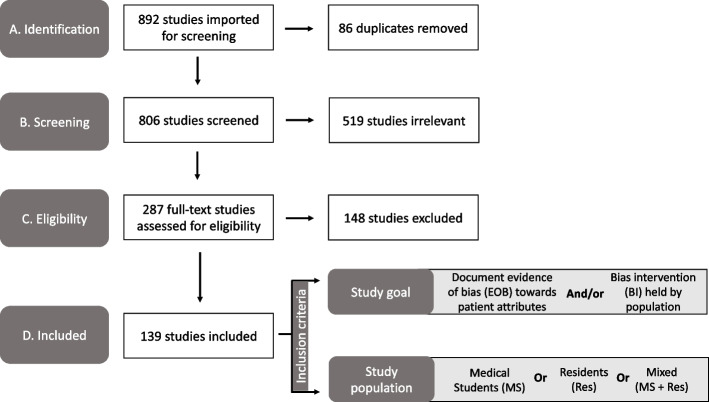
PRISMA diagram of the study selection process used in our scoping review to identify the bias categories that have been reported within medical education literature. Study took place from 2021–2022. Abbreviation: PRISMA, Preferred Reporting Items for Systematic Reviews and Meta-Analyses

Our search strategy yielded a total of 892 unique abstracts which were imported into ‘Covidence’ for screening. A total of 86 duplicate references were removed. Then, 806 titles and abstracts were screened for relevance independently by the authors and 519 studies were excluded at this stage. Any conflicts among the reviewers at this stage were resolved by discussion and referring to the inclusion and exclusion criteria. Then a full text review of the remaining 287 papers was completed by the authors against the inclusion criteria for eligibility. Full text review was also conducted independently by the authors and any conflicts were resolved upon discussion. Finally, we included 139 studies which were used for data extraction (Fig. 1).

### Publication trends in bias research

**Fig. 2:**
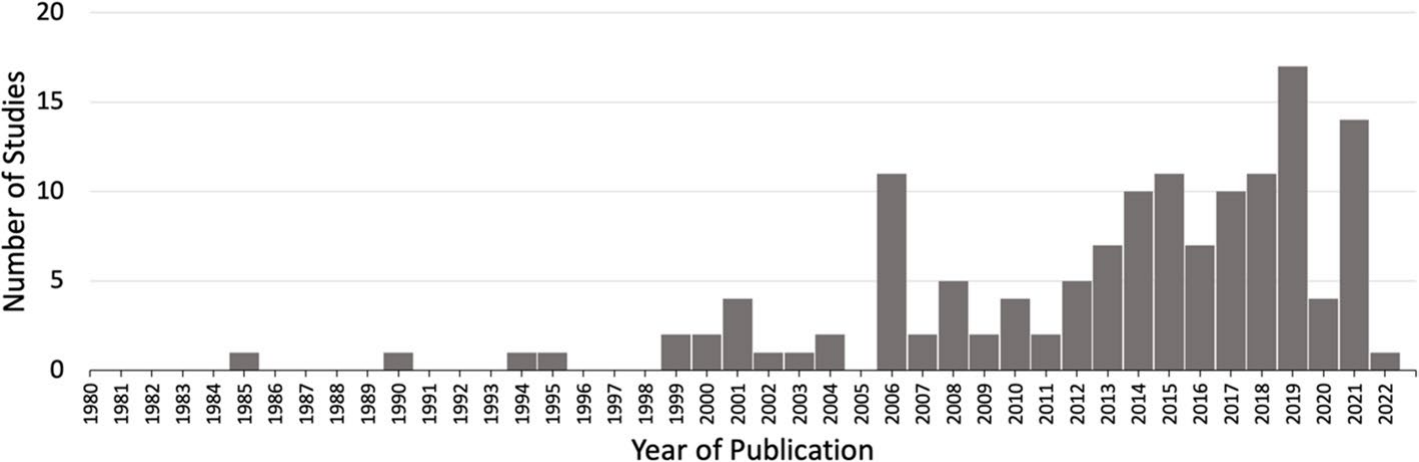
Studies matching inclusion criteria mapped by year of publication. Search criteria included studies addressing bias from 1980–2021 within medical students (MS) or residents (Res) or mixed (MS + Res) populations. ^*^Publication in 2022 was published online ahead of print

First, we charted the studies to demonstrate the timeline of research focused on bias within the study population of our interest (MS or Res or mixed). Our analysis revealed an increase in publications with respect to time (Fig. 2). Of the 139 included studies, fewer studies were published prior to 2001, with a total of only eight papers being published from the years 1985–2000. A substantial increase in publications occurred after 2004, with 2019 being the peak year where most of the studies pertaining to bias were published (Fig. 2).

### Overview of included studies

We present a descriptive analysis of the 139 included studies in Table 1 based on the following parameters: study location, goal of the study, population of the study and the category of bias studied. All of the above parameters except the category of bias included a denominator of 139 studies. Several studies addressed more than one bias characteristic; therefore, we documented 163 biases sorted in 11 categories over the 139 papers. The bias categories that we generated and their respective occurrences are listed in Table 1. Of the 139 studies that were included, most studies originated in the United States (*n* = 89/139, 64%) and Europe (*n* = 20/139, 20%).

**Table 1:** Charting of all included studies fitting our search strategy with references (*n* = 139). Studies mapped based on bias(es) studied may belong to more than one or more category. All other parameters (location, goal, population) contain mutually exclusive criteria

Parameter	No. (%)	References
**Study Location**		
United States	89/139 (64%)	[12, 26–112]
Europe	20/139 (14%)	[11, 113–131]
Asia	9/139 (6%)	[132–140]
Canada	7/139 (5%)	[141–147]
Australia/New Zealand	6/139(4%)	[148–153]
Central/South America	5/139 (4%)	[154–158]
Multi-national	3/139 (2%)	[159–161]
**Goal of Study**		
Document Evidence of Bias (EOB)	69/139 (50%)	[11, 12, 28, 41, 47, 49–52, 54, 56, 61, 64, 66–68, 71, 72, 74, 79, 81, 88, 92, 93, 98–104, 106, 108, 110, 112–116, 119, 120, 122–126, 129–131, 136, 138–143, 145, 148–151, 153–155, 157–159, 161, 162]
Bias Intervention (BI)	51/139 (37%)	[26, 27, 29, 31, 34–36, 39, 40, 43, 45, 46, 48, 55, 57–59, 62, 63, 65, 70, 76–78, 80, 83–87, 89–91, 94–97, 105, 107, 109, 111, 117, 118, 121, 133, 134, 146, 152, 156, 160]
Both (EOB + BI)	19/139 (14%)	[30, 32, 37, 38, 42, 44, 53, 60, 69, 73, 75, 82, 128, 132, 135, 137, 144, 147, 162]
**Population of Study**		
Medical Student (MS)	105/139 (76%)	[12, 26, 27, 29, 33, 35, 37–39, 41, 42, 44–47, 50–56, 58, 59, 61, 64, 66, 68, 69, 71–73, 75–77, 79, 80, 82–86, 88–94, 96–100, 102–115, 117–128, 130, 131, 133, 134, 137, 138, 140, 142, 143, 145, 146, 148–153, 155, 156, 158–162]
Residents (Res)	19/139 (14%)	[32, 34, 36, 43, 48, 60, 62, 65, 78, 87, 95, 116, 132, 135, 141, 144, 147, 154, 157]
Mixed MS [and] Res	15/139 (11%)	[28, 30, 31, 40, 49, 57, 63, 67, 70, 74, 81, 101, 129, 136, 139]
**Category of Bias Studied**		
Race or Ethnicity	39/163 (24%)	[28, 31–36, 39, 43, 45, 48, 51, 52, 60, 62, 65, 68, 69, 71, 85, 87, 97, 98, 102, 107, 109, 110, 118, 128, 131, 141, 142, 148–150, 152, 163–165]
Disease or condition	29/163 (18%)	[11, 67, 82, 86, 93–95, 105, 108–110, 113, 117, 124, 127, 133–135, 138, 142, 143, 146, 147, 154–156, 166, 167]
Weight	22/163 (13%)	[4, 41, 44, 46, 50, 55, 60, 70, 73–75, 98, 101, 103, 104, 120, 121, 130, 137, 151]
LGBTQ +	21/163 (13%)	[29, 30, 49, 58, 60, 61, 78, 92, 94, 95, 98, 100, 110, 122, 123, 125, 126, 136, 140, 142, 158]
Age	16/163 (10%)	[42, 64, 80, 83, 84, 89–91, 96, 106, 112, 115, 157, 162]
Non-Specified	15/163 (9%)	[12, 26, 27, 37, 38, 40, 52–54, 57, 63, 68, 69, 99, 132, 145, 168, 169]
Biological Sex	10/163 (6%)	[72, 81, 114, 116, 137, 139, 142, 153, 159, 161]
Socioeconomic Status	7/163 (4%)	[36, 60, 69, 71, 97, 107, 144, 170]
Education level	2/163 (1%)	[60, 95]
Physical disability	1/163 (1%)	[77]
Rural/Urban	1/163 (1%)	[129]

### Sorting of included research by bias category

We grouped the 139 included studies depending on the patient attribute or the descriptive characteristic against which the bias was studied (Table 1). By sorting the studies into different bias categories, we aimed to not only quantitate the amount of research addressing a particular topic of bias, but also reveal the biases that are understudied.

Through our analysis, we generated 11 descriptive categories against which bias was studied: Age, physical disability, education level, biological sex, disease or condition, LGBTQ + , non-specified, race/ethnicity, rural/urban, socio-economic status, and weight (Table 1). “Age” and “weight” categories included papers that studied bias against older population and higher weight individuals, respectively. The categories “education level” and “socio-economic status” included papers that studied bias against individuals with low education level and individuals belonging to low socioeconomic status, respectively. Within the bias category named ‘biological sex’, we included papers that studied bias against individuals perceived as women/females. Papers that studied bias against gender-identity or sexual orientation were included in its own category named, ‘LGBTQ + ’. The bias category, ‘disease or condition’ was broad and included research on bias against any patient with a specific disease, condition or lifestyle. Studies included in this category researched bias against any physical illnesses, mental illnesses, or sexually transmitted infections. It also included studies that addressed bias against a treatment such as transplant or pain management. It was not significant to report these as individual categories but rather as a whole with a common underlying theme. Rural/urban bias referred to bias that was held against a person based on their place of residence. Studies grouped together in the ‘non-specified bias’ category explored bias without specifying any descriptive characteristic in their methods. These studies did not address any specific bias characteristic in particular but consisted of a study population of our interest (MS or Res or mixed). Based on our analysis, the top five most studied bias categories in our included population within medical education literature were: racial or ethnic bias (*n* = 39/163, 24%), disease or condition bias (*n* = 29/163, 18%), weight bias (*n* = 22/163, 13%), LGBTQ + bias (*n* = 21/163, 13%), and age bias (*n* = 16/163, 10%) which are presented in Table 1.

### Sorting of included research by population

In order to understand the distribution of bias research based on their populations examined, we sorted the included studies in one of the following: medical students (MS), residents (Res) or mixed (Table 1). The following distributions were observed: medical students only (*n* = 105/139, 76%), residents only (*n* = 19/139, 14%) or mixed which consisted of both medical students and residents (*n* = 15/139, 11%). In combination, these results demonstrate that medical educators have focused bias research efforts primarily on medical student populations.

### Sorting of included research by goal

A critical component of this scoping review was to quantify the research goal of the included studies within each of the bias categories. We defined the research goal as either to document evidence of bias (EOB) or to evaluate a bias intervention (BI) (see Fig. 1 for inclusion criteria). Some of the included studies focused on both, documenting evidence in addition to intervening biases and those studies were grouped separately. The analysis revealed that 69/139 (50%) of the included studies focused exclusively on documenting evidence of bias (EOB). There were fewer studies (*n* = 51/139, 37%) which solely focused on bias interventions such as programs, seminars or curricular innovations. A small minority of the included studies were more comprehensive in that they documented EOB followed by an intervention strategy (*n* = 19/139, 11%). These results demonstrate that most bias research is dedicated to documenting evidence of bias among these groups rather than evaluating a bias intervention strategy.

### Research goal distribution

**Fig. 3:**
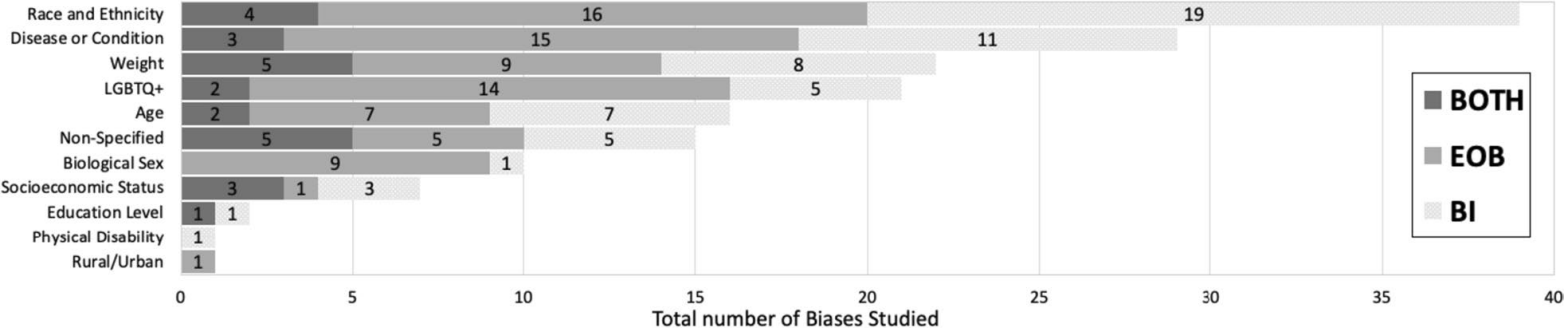
Sorting of total biases (*n* = 163) within medical students or residents or a mixed population based on the *bias category*. Dark grey indicates studies with a dual goal, to document evidence of bias and to intervene bias. Medium grey bars indicate studies which focused on documenting evidence of bias. Light grey bars indicate studies focused on bias intervention within these populations. Numbers inside the bars indicate the total number of biases for the respective study goal. ^*^Non-specified bias includes studies which focused on implicit bias but did not mention the type of bias investigated

Our next objective was to calculate the distribution of studies with respect to the study goal (EOB, BI or both), within the 163 biases studied across the 139 papers as calculated in Table 1. In general, the goal of the studies favors documenting evidence of bias with the exception of race/ethnic bias which is more focused on bias intervention (Fig. 3). Fewer studies were aimed at both, documenting evidence then providing an intervention, across all bias categories.

**Fig. 4:**
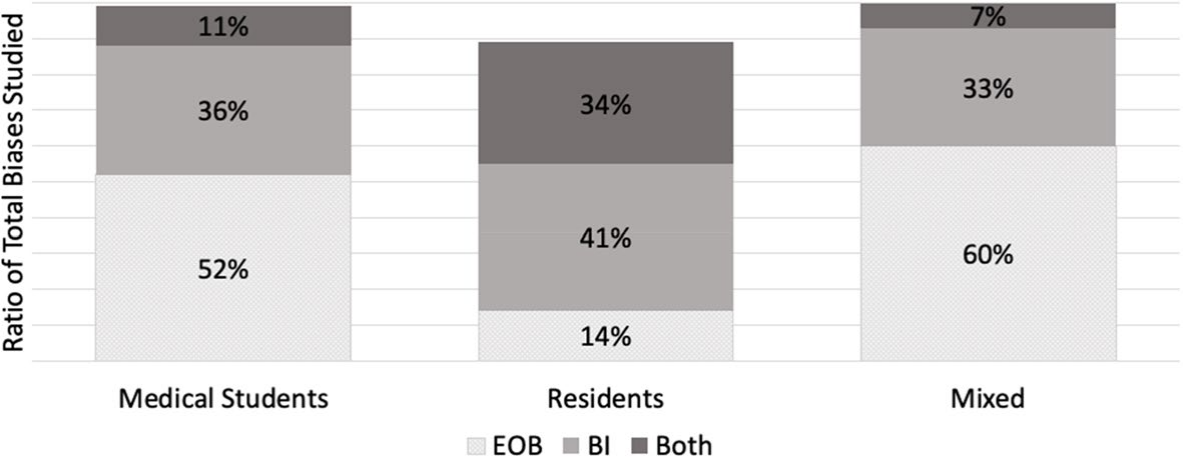
A ratio of the study goal for the total biases (*n* = 163) mapped within each of the study population (MS, Res and Mixed). A study goal with a) documenting evidence of bias (EOB) is depicted in dotted grey, b) bias intervention (BI) in medium grey, and c) a dual focus (EOB + BI) is depicted in dark grey. ^*^N = 122 for medical student studies. ^b^*N* = 26 for residents. ^c^*N* = 15 for mixed

Furthermore, we also calculated the ratio of EOB, BI and both (EOB + BI) within each of our population of interest (MS; *n* = 122, Res; *n* = 26 and mixed; *n* = 15) for the 163 biases observed in our included studies. Over half (*n* = 64/122, 52%) of the total bias occurrences in MS were focused on documenting EOB (Fig. 4). Contrastingly, a shift was observed within resident populations where most biases addressed were aimed at intervention (*n* = 12/26, 41%) rather than EOB (*n* = 4/26, 14%) (Fig. 4). Studies which included both MS and Res (mixed) were primarily focused on documenting EOB (*n* = 9/15, 60%), with 33% (*n* = 5/15) aimed at bias intervention and 7% (*n* = 1/15) which did both (Fig. 4). Although far fewer studies were documented in the Res population it is important to highlight that most of these studies were focused on bias intervention when compared to MS population where we documented a majority of studies focused on evidence of bias.

## Discussion

Addressing biases at an earlier stage of medical career is critical for future physicians engaging with diverse patients, since it is established that bias negatively influences provider-patient interactions [171], clinical decision-making [172] and reduces favorable treatment outcomes [2]. We set out with an intention to explore how bias is addressed within the medical curriculum. Our research question was: how has the trend in bias research changed over time, more specifically a) what is the timeline of papers published? b) what bias characteristics have been studied in the physician-trainee population and c) how are these biases addressed? With the introduction of ‘standards of diversity’ by the Liaison Committee on Medical Education, along with the Association of American Medical Colleges (AAMC) and the American Medical Association (AMA) [173, 174], we certainly expected and observed a sustained uptick in research pertaining to bias. As shown here, research addressing bias in the target population (MS and Res) is on the rise, however only 139 papers fit our inclusion criteria. Of these studies, nearly 90% have been published since 2005 after the “Unequal Treatment: Confronting Racial and Ethnic Disparities in Health Care” report was published in 2003 [7]. However, given the well documented effects of physician held bias, we anticipated significantly more number of studies focused on bias at the medical student or resident level.

A key component from this study was that we generated descriptive categories of biases. Sorting the biases into descriptive categories helps to identify a more targeted approach for a specific bias intervention, rather than to broadly intervene bias as a whole. In fact, our analysis found a number of publications (labeled “non-specified bias” in Table 1) which studied implicit bias without specifying the patient attribute or the characteristic that the bias was against. In total, we generated 11 descriptive categories of bias from our scoping review which are shown in Table 1 and Fig. 3. Furthermore, our bias descriptors grouped similar kinds of biases within a single category. For example, the category, “disease or condition” included papers that studied bias against any type of disease (Mental illness, HIV stigma, diabetes), condition (Pain management), or lifestyle. We neither performed a qualitative assessment of the studies nor did we test the efficacy of the bias intervention studies and consider it a future direction of this work.

Evidence suggests that medical educators and healthcare professionals are struggling to find the appropriate approach to intervene biases [175–177] So far, bias reduction, bias reflection and bias management approaches have been proposed [26, 27, 178]. Previous implicit bias intervention strategies have been shown to be ineffective when biased attitudes of participants were assessed after a lag [179]. Understanding the descriptive categories of bias and previous existing research efforts, as we present here is only a fraction of the challenge. The theory of “cognitive bias” [180] and related branches of research [13, 181–184] have been studied in the field of psychology for over three decades. It is only recently that cognitive bias theory has been applied to the field of medical education medicine, to explain its negative influence on clinical decision-making pertaining only to racial minorities [1, 2, 15–17, 185]. In order to elicit meaningful changes with respect to targeted bias intervention, it is necessary to understand the psychological underpinnings (attitudes) leading to a certain descriptive category of bias (behaviors). The questions which medical educators need to ask are: a) Can these descriptive biases be identified under certain type/s of cognitive errors that elicits the bias and vice versa b) Are we working towards an attitude change which can elicit a sustained positive behavior change among healthcare professionals? And most importantly, c) are we creating a culture where participants voluntarily enroll themselves in bias interventions as opposed to being mandated to participate? Cognitive psychologists and behavioral scientists are well-positioned to help us find answers to these questions as they understand human behavior. Therefore, an interdisciplinary approach, a marriage between cognitive psychologists and medical educators, is key in targeting biases held by medical students, residents, and ultimately future physicians. This review may also be of interest to behavioral psychologists, keen on providing targeted intervening strategies to clinicians depending on the characteristics (age, weight, sex or race) the portrayed bias is against. Further, instead of an individualized approach, we need to strive for systemic changes and evidence-based strategies to intervene biases.

The next element in change is directing intervention strategies at the right stage in clinical education. Our study demonstrated that most of the research collected at the medical student level was focused on documenting evidence of bias. Although the overall number of studies at the resident level were fewer than at the medical student level, the ratio of research in favor of bias intervention was higher at the resident level (see Fig. 3). However, it could be helpful to focus on bias intervention earlier in learning, rather than at a later stage [186]. Additionally, educational resources such as textbooks, preparatory materials, and educators themselves are potential sources of propagating biases and therefore need constant evaluation against best practices [187, 188].

This study has limitations. First, the list of the descriptive bias categories that we generated was not grounded in any particular theory so assigning a category was subjective. Additionally, there were studies that were categorized as “nonspecified” bias as the studies themselves did not mention the specific type of bias that they were addressing. Moreover, we had to exclude numerous publications solely because they were not evidence-based and were either perspectives, commentaries or opinion pieces. Finally, there were overall fewer studies focused on the resident population, so the calculated ratio of MS:Res studies did not compare similar sample sizes.

Future directions of our study include working with behavioral scientists to categorize these bias characteristics (Table 1) into cognitive error types [189]. Additionally, we aim to assess the effectiveness of the intervention strategies and categorize the approach of the intervention strategies.

## Conclusion

The primary goal of our review was to organize, compare and quantify literature pertaining to bias within medical school curricula and residency programs. We neither performed a qualitative assessment of the studies nor did we test the efficacy of studies that were sorted into “bias intervention” as is typical of scoping reviews [22]. In summary, our research identified 11 descriptive categories of biases studied within medical students and resident populations with “race and ethnicity”, “disease or condition”, “weight”, “LGBTQ + ” and “age” being the top five most studied biases. Additionally, we found a greater number of studies conducted in medical students (105/139) when compared to residents (19/139). However, most of the studies in the resident population focused on bias intervention. The results from our review highlight the following gaps: a) bias categories where more research is needed, b) biases that are studied within medical school versus in residency programs and c) study focus in terms of demonstrating the presence of bias or working towards bias intervention.

This review provides a visual analysis of the known categories of bias addressed within the medical school curriculum and in residency programs in addition to providing a comparison of studies with respect to the study goal within medical education literature. The results from our review should be of interest to community organizations, institutions, program directors and medical educators interested in knowing and understanding the types of bias existing within healthcare populations. It might be of special interest to researchers who wish to explore other types of biases that have been understudied within medical school and resident populations, thus filling the gaps existing in bias research.

Despite the number of studies designed to provide bias intervention for MS and Res populations, and an overall cultural shift to be aware of one’s own biases, biases held by both medical students and residents still persist. Further, psychologists have recently demonstrated the ineffectiveness of some bias intervention efforts [179, 190]. Therefore, it is perhaps unrealistic to expect these biases to be eliminated altogether. However, effective intervention strategies grounded in cognitive psychology should be implemented earlier on in medical training. Our focus should be on providing evidence-based approaches and safe spaces for an attitude and culture change, so as to induce actionable behavioral changes.

## Data Availability

The datasets used and/or analyzed during the current study are available from the corresponding author upon reasonable request.

## Abbreviations

MS: Medical student
Res: Resident
EOB: Evidence of bias
BI: Bias intervention

## References

[1] Hagiwara N, Mezuk B, Elston Lafata J, Vrana SR, Fetters MD (2018). Study protocol for investigating physician communication behaviours that link physician implicit racial bias and patient outcomes in Black patients with type 2 diabetes using an exploratory sequential mixed methods design. BMJ Open.

[2] Haider AH, Schneider EB, Sriram N, Dossick DS, Scott VK, Swoboda SM, Losonczy L, Haut ER, Efron DT, Pronovost PJ (2015). Unconscious race and social class bias among acute care surgical clinicians and clinical treatment decisions. JAMA Surg.

[3] Penner LA, Dovidio JF, Gonzalez R, Albrecht TL, Chapman R, Foster T, Harper FW, Hagiwara N, Hamel LM, Shields AF (2016). The effects of oncologist implicit racial bias in racially discordant oncology interactions. J Clin Oncol.

[4] Phelan SM, Burgess DJ, Yeazel MW, Hellerstedt WL, Griffin JM, van Ryn M (2015). Impact of weight bias and stigma on quality of care and outcomes for patients with obesity. Obes Rev.

[5] Garrett SB, Jones L, Montague A, Fa-Yusuf H, Harris-Taylor J, Powell B, Chan E, Zamarripa S, Hooper S, Chambers Butcher BD (2023). Challenges and opportunities for clinician implicit bias training: insights from perinatal care stakeholders. Health Equity.

[6] Shah HS, Bohlen J (2023). Implicit bias. StatPearls.

[7] Institute of Medicine (US) Committee on Understanding and Eliminating Racial and Ethnic Disparities in Health Care. Unequal Treatment: Confronting Racial and Ethnic Disparities in Health Care. In: Smedley BD, Stith AY, Nelson AR, editors. Washington (DC): National Academies Press (US); 2003. PMID: 25032386. 25032386

[8] Dehon E, Weiss N, Jones J, Faulconer W, Hinton E, Sterling S (2017). A systematic review of the impact of physician implicit racial bias on clinical decision making. Acad Emerg Med.

[9] Oliver MN, Wells KM, Joy-Gaba JA, Hawkins CB, Nosek BA (2014). Do physicians' implicit views of African Americans affect clinical decision making?. J Am Board Fam Med.

[10] Rincon-Subtirelu M (2017). Education as a tool to modify anti-obesity bias among pediatric residents. Int J Med Educ.

[11] Gustafsson Sendén M, Renström EA (2019). Gender bias in assessment of future work ability among pain patients - an experimental vignette study of medical students' assessment. Scand J Pain.

[12] Hardeman RR, Burgess D, Phelan S, Yeazel M, Nelson D, van Ryn M (2015). Medical student socio-demographic characteristics and attitudes toward patient centered care: do race, socioeconomic status and gender matter? A report from the medical student CHANGES study. Patient Educ Couns.

[13] Greenwald AG, Banaji MR (1995). Implicit social cognition: attitudes, self-esteem, and stereotypes. Psychol Rev.

[14] Kruse JA, Collins JL, Vugrin M (2022). Educational strategies used to improve the knowledge, skills, and attitudes of health care students and providers regarding implicit bias: an integrative review of the literature. Int J Nurs Stud Adv.

[15] Zestcott CA, Blair IV, Stone J (2016). Examining the presence, consequences, and reduction of implicit bias in health care: a narrative review. Group Process Intergroup Relat.

[16] Hall WJ, Chapman MV, Lee KM, Merino YM, Thomas TW, Payne BK, Eng E, Day SH, Coyne-Beasley T (2015). Implicit racial/ethnic bias among health care professionals and its influence on health care outcomes: a systematic review. Am J Public Health.

[17] FitzGerald C, Hurst S (2017). Implicit bias in healthcare professionals: a systematic review. BMC Med Ethics.

[18] Gonzalez CM, Onumah CM, Walker SA, Karp E, Schwartz R, Lypson ML (2023). Implicit bias instruction across disciplines related to the social determinants of health: a scoping review. Adv Health Sci Educ.

[19] Pham MT, Rajić A, Greig JD, Sargeant JM, Papadopoulos A, McEwen SA (2014). A scoping review of scoping reviews: advancing the approach and enhancing the consistency. Res Synth Methods.

[20] Levac D, Colquhoun H, O'Brien KK (2010). Scoping studies: advancing the methodology. Implement Sci.

[21] Pat C, Geeta S, Sílvia M (2013). Cognitive debiasing 1: origins of bias and theory of debiasing. BMJ Qual Saf.

[22] Arksey H, O'Malley L (2005). Scoping studies: towards a methodological framework. Int J Soc Res Methodol.

[23] Thomas A, Lubarsky S, Durning SJ, Young ME (2017). Knowledge syntheses in medical education: demystifying scoping reviews. Acad Med.

[24] Hagopian A, Thompson MJ, Fordyce M, Johnson KE, Hart LG (2004). The migration of physicians from sub-Saharan Africa to the United States of America: measures of the African brain drain. Hum Resour Health.

[25] Tricco AC, Lillie E, Zarin W, O'Brien KK, Colquhoun H, Levac D, Moher D, Peters MDJ, Horsley T, Weeks L (2018). PRISMA Extension for Scoping Reviews (PRISMA-ScR): checklist and explanation. Ann Intern Med.

[26] Teal CR, Shada RE, Gill AC, Thompson BM, Frugé E, Villarreal GB, Haidet P (2010). When best intentions aren’t enough: Helping medical students develop strategies for managing bias about patients. J Gen Intern Med.

[27] Gonzalez CM, Walker SA, Rodriguez N, Noah YS, Marantz PR (2021). Implicit bias recognition and management in interpersonal encounters and the learning environment: a skills-based curriculum for medical students. MedEdPORTAL.

[28] Hoffman KM, Trawalter S, Axt JR, Oliver MN (2016). Racial bias in pain assessment and treatment recommendations, and false beliefs about biological differences between blacks and whites. Proc Natl Acad Sci U S A.

[29] Mayfield JJ, Ball EM, Tillery KA, Crandall C, Dexter J, Winer JM, Bosshardt ZM, Welch JH, Dolan E, Fancovic ER (2017). Beyond men, women, or both: a comprehensive, LGBTQ-inclusive, implicit-bias-aware, standardized-patient-based sexual history taking curriculum. MedEdPORTAL.

[30] Morris M, Cooper RL, Ramesh A, Tabatabai M, Arcury TA, Shinn M, Im W, Juarez P, Matthews-Juarez P (2019). Training to reduce LGBTQ-related bias among medical, nursing, and dental students and providers: a systematic review. BMC Med Educ.

[31] Perdomo J, Tolliver D, Hsu H, He Y, Nash KA, Donatelli S, Mateo C, Akagbosu C, Alizadeh F, Power-Hays A (2019). Health equity rounds: an interdisciplinary case conference to address implicit bias and structural racism for faculty and trainees. MedEdPORTAL.

[32] Sherman MD, Ricco J, Nelson SC, Nezhad SJ, Prasad S (2019). Implicit bias training in a residency program: aiming for enduring effects. Fam Med.

[33] van Ryn M, Hardeman R, Phelan SM, Burgess DJ, Dovidio JF, Herrin J, Burke SE, Nelson DB, Perry S, Yeazel M (2015). Medical school experiences associated with change in implicit racial bias among 3547 students: a medical student CHANGES study report. J Gen Intern Med.

[34] Chary AN, Molina MF, Dadabhoy FZ, Manchanda EC (2020). Addressing racism in medicine through a resident-led health equity retreat. West J Emerg Med.

[35] DallaPiazza M, Padilla-Register M, Dwarakanath M, Obamedo E, Hill J, Soto-Greene ML (2018). Exploring racism and health: an intensive interactive session for medical students. MedEdPORTAL.

[36] Dennis SN, Gold RS, Wen FK (2019). Learner reactions to activities exploring racism as a social determinant of health. Fam Med.

[37] Gonzalez CM, Walker SA, Rodriguez N, Karp E, Marantz PR (2020). It can be done! a skills-based elective in implicit bias recognition and management for preclinical medical students. Acad Med.

[38] Motzkus C, Wells RJ, Wang X, Chimienti S, Plummer D, Sabin J, Allison J, Cashman S (2019). Pre-clinical medical student reflections on implicit bias: Implications for learning and teaching. PLoS ONE.

[39] Phelan SM, Burke SE, Cunningham BA, Perry SP, Hardeman RR, Dovidio JF, Herrin J, Dyrbye LN, White RO, Yeazel MW (2019). The effects of racism in medical education on students’ decisions to practice in underserved or minority communities. Acad Med.

[40] Zeidan A, Tiballi A, Woodward M, Di Bartolo IM (2019). Targeting implicit bias in medicine: lessons from art and archaeology. West J Emerg Med.

[41] Baker TK, Smith GS, Jacobs NN, Houmanfar R, Tolles R, Kuhls D, Piasecki M (2017). A deeper look at implicit weight bias in medical students. Adv Health Sci Educ Theory Pract.

[42] Eymard AS, Douglas DH (2012). Ageism among health care providers and interventions to improve their attitudes toward older adults: an integrative review. J Gerontol Nurs.

[43] Garrison CB, McKinney-Whitson V, Johnston B, Munroe A (2018). Race matters: addressing racism as a health issue. Int J Psychiatry Med.

[44] Geller G, Watkins PA (2018). Addressing medical students’ negative bias toward patients with obesity through ethics education. AMA J Ethics.

[45] Onyeador IN, Wittlin NM, Burke SE, Dovidio JF, Perry SP, Hardeman RR, Dyrbye LN, Herrin J, Phelan SM, van Ryn M (2020). The value of interracial contact for reducing anti-black bias among non-black physicians: a Cognitive Habits and Growth Evaluation (CHANGE) study report. Psychol Sci.

[46] Poustchi Y, Saks NS, Piasecki AK, Hahn KA, Ferrante JM (2013). Brief intervention effective in reducing weight bias in medical students. Fam Med.

[47] Ruiz JG, Andrade AD, Anam R, Taldone S, Karanam C, Hogue C, Mintzer MJ (2015). Group-based differences in anti-aging bias among medical students. Gerontol Geriatr Educ.

[48] Simpson T, Evans J, Goepfert A, Elopre L (2022). Implementing a graduate medical education anti-racism workshop at an academic university in the Southern USA. Med Educ Online.

[49] Wittlin NM, Dovidio JF, Burke SE, Przedworski JM, Herrin J, Dyrbye L, Onyeador IN, Phelan SM, van Ryn M (2019). Contact and role modeling predict bias against lesbian and gay individuals among early-career physicians: a longitudinal study. Soc Sci Med.

[50] Miller DP, Spangler JG, Vitolins MZ, Davis SW, Ip EH, Marion GS, Crandall SJ (2013). Are medical students aware of their anti-obesity bias?. Acad Med.

[51] Gonzalez CM, Deno ML, Kintzer E, Marantz PR, Lypson ML, McKee MD (2019). A qualitative study of New York medical student views on implicit bias instruction: implications for curriculum development. J Gen Intern Med.

[52] Gonzalez CM, Kim MY, Marantz PR (2014). Implicit bias and its relation to health disparities: a teaching program and survey of medical students. Teach Learn Med.

[53] Gonzalez CM, Nava S, List J, Liguori A, Marantz PR (2021). How assumptions and preferences can affect patient care: an introduction to implicit bias for first-year medical students. MedEdPORTAL.

[54] Hernandez RA, Haidet P, Gill AC, Teal CR (2013). Fostering students' reflection about bias in healthcare: cognitive dissonance and the role of personal and normative standards. Med Teach.

[55] Kushner RF, Zeiss DM, Feinglass JM, Yelen M (2014). An obesity educational intervention for medical students addressing weight bias and communication skills using standardized patients. BMC Med Educ.

[56] Nazione S, Silk KJ (2013). Patient race and perceived illness responsibility: effects on provider helping and bias. Med Educ.

[57] Ogunyemi D (2021). Defeating unconscious bias: the role of a structured, reflective, and interactive workshop. J Grad Med Educ.

[58] Phelan SM, Burke SE, Hardeman RR, White RO, Przedworski J, Dovidio JF, Perry SP, Plankey M, A Cunningham B, Finstad D (2017). Medical school factors associated with changes in implicit and explicit bias against gay and lesbian people among 3492 graduating medical students. J Gen Intern Med.

[59] Phelan SM, Puhl RM, Burke SE, Hardeman R, Dovidio JF, Nelson DB, Przedworski J, Burgess DJ, Perry S, Yeazel MW (2015). The mixed impact of medical school on medical students’ implicit and explicit weight bias. Med Educ.

[60] Barber Doucet H, Ward VL, Johnson TJ, Lee LK (2021). Implicit bias and caring for diverse populations: pediatric trainee attitudes and gaps in training. Clin Pediatr (Phila).

[61] Burke SE, Dovidio JF, Przedworski JM, Hardeman RR, Perry SP, Phelan SM, Nelson DB, Burgess DJ, Yeazel MW, van Ryn M (2015). Do contact and empathy mitigate bias against gay and lesbian people among heterosexual first-year medical students? A report from the medical student CHANGE study. Acad Med.

[62] Johnston B, McKinney-Whitson V, Garrison V (2021). Race matters: addressing racism as a health issue. WMJ.

[63] Kost A, Akande T, Jones R, Gabert R, Isaac M, Dettmar NS (2021). Use of patient identifiers at the University of Washington School of Medicine: building institutional consensus to reduce bias and stigma. Fam Med.

[64] Madan AK, Aliabadi-Wahle S, Beech DJ (2001). Ageism in medical students’ treatment recommendations: the example of breast-conserving procedures. Acad Med.

[65] Marbin J, Lewis L, Kuo AK, Schudel C, Gutierrez JR (2021). The power of place: travel to explore structural racism and health disparities. Acad Med.

[66] Phelan SM, Dovidio JF, Puhl RM, Burgess DJ, Nelson DB, Yeazel MW, Hardeman R, Perry S, van Ryn M (2014). Implicit and explicit weight bias in a national sample of 4,732 medical students: the medical student CHANGES study. Obesity (Silver Spring).

[67] Van J, Aloman C, Reau N (2021). Potential bias and misconceptions in liver transplantation for alcohol- and obesity-related liver disease. Am J Gastroenterol.

[68] White-Means S, Zhiyong D, Hufstader M, Brown LT (2009). Cultural competency, race, and skin tone bias among pharmacy, nursing, and medical students: implications for addressing health disparities. Med Care Res Rev.

[69] Williams RL, Vasquez CE, Getrich CM, Kano M, Boursaw B, Krabbenhoft C, Sussman AL (2018). Racial/gender biases in student clinical decision-making: a mixed-method study of medical school attributes associated with lower incidence of biases. J Gen Intern Med.

[70] Cohen RW, Persky S (2019). Influence of weight etiology information and trainee characteristics on physician-trainees’ clinical and interpersonal communication. Patient Educ Couns.

[71] Haider AH, Sexton J, Sriram N, Cooper LA, Efron DT, Swoboda S, Villegas CV, Haut ER, Bonds M, Pronovost PJ (2011). Association of unconscious race and social class bias with vignette-based clinical assessments by medical students. JAMA.

[72] Lewis R, Lamdan RM, Wald D, Curtis M (2006). Gender bias in the diagnosis of a geriatric standardized patient: a potential confounding variable. Acad Psychiatry.

[73] Matharu K, Shapiro JF, Hammer RR, Kravitz RL, Wilson MD, Fitzgerald FT (2014). Reducing obesity prejudice in medical education. Educ Health.

[74] McLean ME, McLean LE, McLean-Holden AC, Campbell LF, Horner AM, Kulkarni ML, Melville LD, Fernandez EA (2021). Interphysician weight bias: a cross-sectional observational survey study to guide implicit bias training in the medical workplace. Acad Emerg Med.

[75] Meadows A, Higgs S, Burke SE, Dovidio JF, van Ryn M, Phelan SM (2017). Social dominance orientation, dispositional empathy, and need for cognitive closure moderate the impact of empathy-skills training, but not patient contact, on medical students’ negative attitudes toward higher-weight patients. Front Psychol.

[76] Stone J, Moskowitz GB, Zestcott CA, Wolsiefer KJ (2020). Testing active learning workshops for reducing implicit stereotyping of Hispanics by majority and minority group medical students. Stigma Health.

[77] Symons AB, Morley CP, McGuigan D, Akl EA (2014). A curriculum on care for people with disabilities: effects on medical student self-reported attitudes and comfort level. Disabil Health J.

[78] Ufomata E, Eckstrand KL, Hasley P, Jeong K, Rubio D, Spagnoletti C (2018). Comprehensive internal medicine residency curriculum on primary care of patients who identify as LGBT. LGBT Health.

[79] Aultman JM, Borges NJ (2006). A clinical and ethical investigation of pre-medical and medical students' attitudes, knowledge, and understanding of HIV. Med Educ Online.

[80] Bates T, Cohan M, Bragg DS, Bedinghaus J (2006). The Medical College of Wisconsin senior mentor program: experience of a lifetime. Gerontol Geriatr Educ.

[81] Chiaramonte GR, Friend R (2006). Medical students' and residents' gender bias in the diagnosis, treatment, and interpretation of coronary heart disease symptoms. Health Psychol.

[82] Friedberg F, Sohl SJ, Halperin PJ (2008). Teaching medical students about medically unexplained illnesses: a preliminary study. Med Teach.

[83] Gonzales E, Morrow-Howell N, Gilbert P (2010). Changing medical students' attitudes toward older adults. Gerontol Geriatr Educ.

[84] Hinners CK, Potter JF (2006). A partnership between the University of Nebraska College of Medicine and the community: fostering positive attitudes towards the aged. Gerontol Geriatr Educ.

[85] Lee M, Coulehan JL (2006). Medical students’ perceptions of racial diversity and gender equality. Med Educ.

[86] Schmetzer AD, Lafuze JE (2008). Overcoming stigma: involving families in medical student and psychiatric residency education. Acad Psychiatry.

[87] Willen SS, Bullon A, Good MJD (2010). Opening up a huge can of worms: reflections on a “cultural sensitivity” course for psychiatry residents. Harv Rev Psychiatry.

[88] Dogra N, Karnik N (2003). First-year medical students' attitudes toward diversity and its teaching: an investigation at one U.S. medical school. Acad Med.

[89] Fitzpatrick C, Musser A, Mosqueda L, Boker J, Prislin M (2006). Student senior partnership program: University of California Irvine School of Medicine. Gerontol Geriatr Educ.

[90] Hoffman KG, Gray P, Hosokawa MC, Zweig SC (2006). Evaluating the effectiveness of a senior mentor program: the University of Missouri-Columbia School of Medicine. Gerontol Geriatr Educ.

[91] Kantor BS, Myers MR (2006). From aging…to saging-the Ohio State Senior Partners Program: longitudinal and experiential geriatrics education. Gerontol Geriatr Educ.

[92] Klamen DL, Grossman LS, Kopacz DR (1999). Medical student homophobia. J Homosex.

[93] Kopacz DR, Grossman LS, Klamen DL (1999). Medical students and AIDS: knowledge, attitudes and implications for education. Health Educ Res.

[94] Leiblum SR (2001). An established medical school human sexuality curriculum: description and evaluation. Sex Relatsh Ther.

[95] Rastegar DA, Fingerhood MI, Jasinski DR (2004). A resident clerkship that combines inpatient and outpatient training in substance abuse and HIV care. Subst Abuse.

[96] Roberts E, Richeson NA, Thornhill JTIV, Corwin SJ, Eleazer GP (2006). The senior mentor program at the University of South Carolina School of Medicine: an innovative geriatric longitudinal curriculum. Gerontol Geriatr Educ.

[97] Burgess DJ, Burke SE, Cunningham BA, Dovidio JF, Hardeman RR, Hou YF, Nelson DB, Perry SP, Phelan SM, Yeazel MW (2016). Medical students’ learning orientation regarding interracial interactions affects preparedness to care for minority patients: a report from medical student CHANGES. BMC Med Educ.

[98] Burgess DJ, Hardeman RR, Burke SE, Cunningham BA, Dovidio JF, Nelson DB, Perry SP, Phelan SM, Yeazel MW, Herrin J (2019). Incoming medical students’ political orientation affects outcomes related to care of marginalized groups: results from the medical student CHANGES study. J Health Pol Policy Law.

[99] Kurtz ME, Johnson SM, Tomlinson T, Fiel NJ (1985). Teaching medical students the effects of values and stereotyping on the doctor/patient relationship. Soc Sci Med.

[100] Matharu K, Kravitz RL, McMahon GT, Wilson MD, Fitzgerald FT (2012). Medical students’ attitudes toward gay men. BMC Med Educ.

[101] Pearl RL, Argueso D, Wadden TA (2017). Effects of medical trainees' weight-loss history on perceptions of patients with obesity. Med Educ.

[102] Perry SP, Dovidio JF, Murphy MC, van Ryn M (2015). The joint effect of bias awareness and self-reported prejudice on intergroup anxiety and intentions for intergroup contact. Cultur Divers Ethnic Minor Psychol.

[103] Phelan SM, Burgess DJ, Burke SE, Przedworski JM, Dovidio JF, Hardeman R, Morris M, van Ryn M (2015). Beliefs about the causes of obesity in a national sample of 4th year medical students. Patient Educ Couns.

[104] Phelan SM, Puhl RM, Burgess DJ, Natt N, Mundi M, Miller NE, Saha S, Fischer K, van Ryn M (2021). The role of weight bias and role-modeling in medical students’ patient-centered communication with higher weight standardized patients. Patient Educ Couns.

[105] Polan HJ, Auerbach MI, Viederman M (1990). AIDS as a paradigm of human behavior in disease: impact and implications of a course. Acad Psychiatry.

[106] Reuben DB, Fullerton JT, Tschann JM, Croughan-Minihane M (1995). Attitudes of beginning medical students toward older persons: a five-campus study. J Am Geriatr Soc.

[107] Tsai J (2021). Building structural empathy to marshal critical education into compassionate practice: evaluation of a medical school critical race theory course. J Law Med Ethics.

[108] Weyant RJ, Bennett ME, Simon M, Palaisa J (1994). Desire to treat HIV-infected patients: similarities and differences across health-care professions. AIDS.

[109] Ross PT, Lypson ML (2014). Using artistic-narrative to stimulate reflection on physician bias. Teach Learn Med.

[110] Calabrese SK, Earnshaw VA, Krakower DS, Underhill K, Vincent W, Magnus M, Hansen NB, Kershaw TS, Mayer KH, Betancourt JR (2018). A closer look at racism and heterosexism in medical students' clinical decision-making related to HIV Pre-Exposure Prophylaxis (PrEP): implications for PrEP education. AIDS Behav.

[111] Fitterman-Harris HF, Vander Wal JS (2021). Weight bias reduction among first-year medical students: a quasi-randomized, controlled trial. Clin Obes.

[112] Madan AK, Cooper L, Gratzer A, Beech DJ (2006). Ageism in breast cancer surgical options by medical students. Tenn Med.

[113] Bikmukhametov DA, Anokhin VA, Vinogradova AN, Triner WR, McNutt LA (2012). Bias in medicine: a survey of medical student attitudes towards HIV-positive and marginalized patients in Russia, 2010. J Int AIDS Soc.

[114] Dijkstra AF, Verdonk P, Lagro-Janssen AL (2008). Gender bias in medical textbooks: examples from coronary heart disease, depression, alcohol abuse and pharmacology. Med Educ.

[115] Dobrowolska B, Jędrzejkiewicz B, Pilewska-Kozak A, Zarzycka D, Ślusarska B, Deluga A, Kościołek A, Palese A (2019). Age discrimination in healthcare institutions perceived by seniors and students. Nurs Ethics.

[116] Hamberg K, Risberg G, Johansson EE, Westman G (2002). Gender bias in physicians’ management of neck pain: a study of the answers in a Swedish national examination. J Womens Health Gend Based Med.

[117] Magliano L, Read J, Sagliocchi A, Oliviero N, D'Ambrosio A, Campitiello F, Zaccaro A, Guizzaro L, Patalano M (2014). “Social dangerousness and incurability in schizophrenia”: results of an educational intervention for medical and psychology students. Psychiatry Res.

[118] Reis SP, Wald HS (2015). Contemplating medicine during the Third Reich: scaffolding professional identity formation for medical students. Acad Med.

[119] Schroyen S, Adam S, Marquet M, Jerusalem G, Thiel S, Giraudet AL, Missotten P (2018). Communication of healthcare professionals: Is there ageism?. Eur J Cancer Care (Engl).

[120] Swift JA, Hanlon S, El-Redy L, Puhl RM, Glazebrook C (2013). Weight bias among UK trainee dietitians, doctors, nurses and nutritionists. J Hum Nutr Diet.

[121] Swift JA, Tischler V, Markham S, Gunning I, Glazebrook C, Beer C, Puhl R (2013). Are anti-stigma films a useful strategy for reducing weight bias among trainee healthcare professionals? Results of a pilot randomized control trial. Obes Facts.

[122] Yertutanol FDK, Candansayar S, Seydaoğlu G (2019). Homophobia in health professionals in Ankara, Turkey: developing a scale. Transcult Psychiatry.

[123] Arnold O, Voracek M, Musalek M, Springer-Kremser M (2004). Austrian medical students’ attitudes towards male and female homosexuality: a comparative survey. Wien Klin Wochenschr.

[124] Arvaniti A, Samakouri M, Kalamara E, Bochtsou V, Bikos C, Livaditis M (2009). Health service staff’s attitudes towards patients with mental illness. Soc Psychiatry Psychiatr Epidemiol.

[125] Lopes L, Gato J, Esteves M (2016). Portuguese medical students’ knowledge and attitudes towards homosexuality. Acta Med Port.

[126] Papadaki V, Plotnikof K, Gioumidou M, Zisimou V, Papadaki E (2015). A comparison of attitudes toward lesbians and gay men among students of helping professions in Crete, Greece: the cases of social work, psychology, medicine, and nursing. J Homosex.

[127] Papaharitou S, Nakopoulou E, Moraitou M, Tsimtsiou Z, Konstantinidou E, Hatzichristou D (2008). Exploring sexual attitudes of students in health professions. J Sex Med.

[128] Roberts JH, Sanders T, Mann K, Wass V (2010). Institutional marginalisation and student resistance: barriers to learning about culture, race and ethnicity. Adv Health Sci Educ.

[129] Wilhelmi L, Ingendae F, Steinhaeuser J (2018). What leads to the subjective perception of a ‘rural area’? A qualitative study with undergraduate students and postgraduate trainees in Germany to tailor strategies against physician's shortage. Rural Remote Health.

[130] Herrmann-Werner A, Loda T, Wiesner LM, Erschens RS, Junne F, Zipfel S (2019). Is an obesity simulation suit in an undergraduate medical communication class a valuable teaching tool? A cross-sectional proof of concept study. BMJ Open.

[131] Ahadinezhad B, Khosravizadeh O, Maleki A, Hashtroodi A. Implicit racial bias among medical graduates and students by an IAT measure: a systematic review and meta-analysis. Ir J Med Sci. 2022;191(4):1941–9. 10.1007/s11845-021-02756-3. 10.1007/s11845-021-02756-3 34495481

[132] Hsieh JG, Hsu M, Wang YW (2016). An anthropological approach to teach and evaluate cultural competence in medical students - the application of mini-ethnography in medical history taking. Med Educ Online.

[133] Poreddi V, Thimmaiah R, Math SB (2015). Attitudes toward people with mental illness among medical students. J Neurosci Rural Pract.

[134] Mino Y, Yasuda N, Tsuda T, Shimodera S (2001). Effects of a one-hour educational program on medical students’ attitudes to mental illness. Psychiatry Clin Neurosci.

[135] Omori A, Tateno A, Ideno T, Takahashi H, Kawashima Y, Takemura K, Okubo Y (2012). Influence of contact with schizophrenia on implicit attitudes towards schizophrenia patients held by clinical residents. BMC Psychiatry.

[136] Banwari G, Mistry K, Soni A, Parikh N, Gandhi H (2015). Medical students and interns’ knowledge about and attitude towards homosexuality. J Postgrad Med.

[137] Lee SY (2018). Obesity education in medical school curricula in Korea. J Obes Metab Syndr.

[138] Aruna G, Mittal S, Yadiyal MB, Acharya C, Acharya S, Uppulari C (2016). Perception, knowledge, and attitude toward mental disorders and psychiatry among medical undergraduates in Karnataka: a cross-sectional study. Indian J Psychiatry.

[139] Wong YL (2009). Review paper: gender competencies in the medical curriculum: addressing gender bias in medicine. Asia Pac J Public Health.

[140] Earnshaw VA, Jin H, Wickersham JA, Kamarulzaman A, John J, Lim SH, Altice FL (2016). Stigma toward men who have sex with men among future healthcare providers in Malaysia: would more interpersonal contact reduce prejudice?. AIDS Behav.

[141] Larson B, Herx L, Williamson T, Crowshoe L (2011). Beyond the barriers: family medicine residents’ attitudes towards providing Aboriginal health care. Med Educ.

[142] Wagner AC, Girard T, McShane KE, Margolese S, Hart TA (2017). HIV-related stigma and overlapping stigmas towards people living with HIV among health care trainees in Canada. AIDS Educ Prev.

[143] Tellier P-P, Bélanger E, Rodríguez C, Ware MA, Posel N (2013). Improving undergraduate medical education about pain assessment and management: a qualitative descriptive study of stakeholders’ perceptions. Pain Res Manage.

[144] Loignon C, Boudreault-Fournier A, Truchon K, Labrousse Y, Fortin B (2014). Medical residents reflect on their prejudices toward poverty: a photovoice training project. BMC Med Educ.

[145] Phillips SP, Clarke M (2012). More than an education: the hidden curriculum, professional attitudes and career choice. Med Educ.

[146] Jaworsky D, Gardner S, Thorne JG, Sharma M, McNaughton N, Paddock S, Chew D, Lees R, Makuwaza T, Wagner A (2017). The role of people living with HIV as patient instructors—Reducing stigma and improving interest around HIV care among medical students. AIDS Care.

[147] Sukhera J, Wodzinski M, Teunissen PW, Lingard L, Watling C (2018). Striving while accepting: exploring the relationship between identity and implicit bias recognition and management. Acad Med.

[148] Harris R, Cormack D, Curtis E, Jones R, Stanley J, Lacey C (2016). Development and testing of study tools and methods to examine ethnic bias and clinical decision-making among medical students in New Zealand: the Bias and Decision-Making in Medicine (BDMM) study. BMC Med Educ.

[149] Cormack D, Harris R, Stanley J, Lacey C, Jones R, Curtis E (2018). Ethnic bias amongst medical students in Aotearoa/New Zealand: findings from the Bias and Decision Making in Medicine (BDMM) study. PLoS ONE.

[150] Harris R, Cormack D, Stanley J, Curtis E, Jones R, Lacey C (2018). Ethnic bias and clinical decision-making among New Zealand medical students: an observational study. BMC Med Educ.

[151] Robinson EL, Ball LE, Leveritt MD (2014). Obesity bias among health and non-health students attending an Australian university and their perceived obesity education. J Nutr Educ Behav.

[152] Sopoaga F, Zaharic T, Kokaua J, Covello S (2017). Training a medical workforce to meet the needs of diverse minority communities. BMC Med Educ.

[153] Parker R, Larkin T, Cockburn J (2017). A visual analysis of gender bias in contemporary anatomy textbooks. Soc Sci Med.

[154] Gomes MdM (2000). Doctors’ perspectives and practices regarding epilepsy. Arq Neuropsiquiatr.

[155] Caixeta J, Fernandes PT, Bell GS, Sander JW, Li LM (2007). Epilepsy perception amongst university students - A survey. Arq Neuropsiquiatr.

[156] Tedrus GMAS, Fonseca LC, da Câmara Vieira AL (2007). Knowledge and attitudes toward epilepsy amongst students in the health area: intervention aimed at enlightenment. Arq Neuropsiquiatr.

[157] Gomez-Moreno C, Verduzco-Aguirre H, Contreras-Garduño S, Perez-de-Acha A, Alcalde-Castro J, Chavarri-Guerra Y, García-Lara JMA, Navarrete-Reyes AP, Avila-Funes JA, Soto-Perez-de-Celis E (2019). Perceptions of aging and ageism among Mexican physicians-in-training. Clin Transl Oncol.

[158] Campbell MH, Gromer J, Emmanuel MK, Harvey A. Attitudes Toward Transgender People Among Future Caribbean Doctors. Arch Sex Behav. 2022;51(4):1903-11. 10.1007/s10508-021-02205-3. 10.1007/s10508-021-02205-3 34782942

[159] Hatala R, Case SM (2000). Examining the influence of gender on medical students' decision making. J Womens Health Gend Based Med.

[160] Deb T, Lempp H, Bakolis I, et al. Responding to experienced and anticipated discrimination (READ): anti -stigma training for medical students towards patients with mental illness – study protocol for an international multisite non-randomised controlled study. BMC Med Educ. 2019;19:41. 10.1186/s12909-019-1472-7. 10.1186/s12909-019-1472-7 PMC6357462 30704531

[161] Morgan S, Plaisant O, Lignier B, Moxham BJ (2014). Sexism and anatomy, as discerned in textbooks and as perceived by medical students at Cardiff University and University of Paris Descartes. J Anat.

[162] Alford CL, Miles T, Palmer R, Espino D (2001). An introduction to geriatrics for first-year medical students. J Am Geriatr Soc.

[163] Stone J, Moskowitz GB (2011). Non-conscious bias in medical decision making: what can be done to reduce it?. Med Educ.

[164] Nazione S (2015). Slimming down medical provider weight bias in an obese nation. Med Educ.

[165] Dogra N, Connin S, Gill P, Spencer J, Turner M (2005). Teaching of cultural diversity in medical schools in the United Kingdom and Republic of Ireland: cross sectional questionnaire survey. BMJ.

[166] Aultman JM, Borges NJ (2006). A clinical and ethical investigation of pre-medical and medical students' attitudes, knowledge, and understanding of HIV. Med Educ Online.

[167] Deb T, Lempp H, Bakolis I, Vince T, Waugh W, Henderson C, Thornicroft G, Ando S, Yamaguchi S, Matsunaga A (2019). Responding to experienced and anticipated discrimination (READ): anti -stigma training for medical students towards patients with mental illness – study protocol for an international multisite non-randomised controlled study. BMC Med Educ.

[168] Gonzalez CM, Grochowalski JH, Garba RJ, Bonner S, Marantz PR (2021). Validity evidence for a novel instrument assessing medical student attitudes toward instruction in implicit bias recognition and management. BMC Med Educ.

[169] Ogunyemi D (2021). A practical approach to implicit bias training. J Grad Med Educ.

[170] Dennis GC (2001). Racism in medicine: planning for the future. J Natl Med Assoc.

[171] Maina IW, Belton TD, Ginzberg S, Singh A, Johnson TJ (2018). A decade of studying implicit racial/ethnic bias in healthcare providers using the implicit association test. Soc Sci Med.

[172] Blair IV, Steiner JF, Hanratty R, Price DW, Fairclough DL, Daugherty SL, Bronsert M, Magid DJ, Havranek EP (2014). An investigation of associations between clinicians’ ethnic or racial bias and hypertension treatment, medication adherence and blood pressure control. J Gen Intern Med.

[173] Stanford FC (2020). The importance of diversity and inclusion in the healthcare workforce. J Natl Med Assoc.

[174] Education LCoM (2009). Standards on diversity.

[175] Onyeador IN, Hudson STJ, Lewis NA (2021). Moving beyond implicit bias training: policy insights for increasing organizational diversity. Policy Insights Behav Brain Sci.

[176] Forscher PS, Mitamura C, Dix EL, Cox WTL, Devine PG (2017). Breaking the prejudice habit: mechanisms, timecourse, and longevity. J Exp Soc Psychol.

[177] Lai CK, Skinner AL, Cooley E, Murrar S, Brauer M, Devos T, Calanchini J, Xiao YJ, Pedram C, Marshburn CK (2016). Reducing implicit racial preferences: II. Intervention effectiveness across time. J Exp Psychol Gen.

[178] Sukhera J, Watling CJ, Gonzalez CM (2020). Implicit bias in health professions: from recognition to transformation. Acad Med.

[179] Vuletich HA, Payne BK (2019). Stability and change in implicit bias. Psychol Sci.

[180] Tversky A, Kahneman D (1974). Judgment under uncertainty: Heuristics and biases. Science.

[181] Miller DT, Ross M (1975). Self-serving biases in the attribution of causality: fact or fiction?. Psychol Bull.

[182] Nickerson RS (1998). Confirmation bias: a ubiquitous phenomenon in many guises. Rev Gen Psychol.

[183] Suveren Y (2022). Unconscious bias: definition and significance. Psikiyatride Guncel Yaklasimlar.

[184] Dietrich D, Olson M (1993). A demonstration of hindsight bias using the Thomas confirmation vote. Psychol Rep.

[185] Green AR, Carney DR, Pallin DJ, Ngo LH, Raymond KL, Iezzoni LI, Banaji MR (2007). Implicit bias among physicians and its prediction of thrombolysis decisions for black and white patients. J Gen Intern Med.

[186] Rushmer R, Davies HT (2004). Unlearning in health care. Qual Saf Health Care.

[187] Vu MT, Pham TTT. Gender, critical pedagogy, and textbooks: Understanding teachers’ (lack of) mediation of the hidden curriculum in the EFL classroom. Lang Teach Res. 2022;0(0). 10.1177/13621688221136937.

[188] Kalantari A, Alvarez A, Battaglioli N, Chung A, Cooney R, Boehmer SJ, Nwabueze A, Gottlieb M (2022). Sex and race visual representation in emergency medicine textbooks and the hidden curriculum. AEM Educ Train.

[189] Satya-Murti S, Lockhart J (2015). Recognizing and reducing cognitive bias in clinical and forensic neurology. Neurol Clin Pract.

[190] Chang EH, Milkman KL, Gromet DM, Rebele RW, Massey C, Duckworth AL, Grant AM (2019). The mixed effects of online diversity training. Proc Natl Acad Sci U S A.

